# Integrating Guilt and Shame into the Self-Concept: The Influence of Future Opportunities

**DOI:** 10.3390/bs14060472

**Published:** 2024-06-03

**Authors:** Hyeman Choi

**Affiliations:** Department of Psychology, Gachon University, Seongnam 13120, Republic of Korea; choih@gachon.ac.kr; Tel.: +82-31-750-2663

**Keywords:** self-conscious emotion, shame, guilt, self-integration, identity integration, self-concept, intrapersonal learning

## Abstract

This study explored the integration of guilt and shame experiences into the self-concept, focusing on how perceived future opportunities affect this process. The participants in Study 1 (*N* = 201) and Study 2 (*N* = 221) recalled experiences that elicited either guilt or shame and that they believed could occur again in the future (i.e., repeatable) or could not (i.e., non-repeatable). The results showed that when the participants viewed an event as repeatable, suggesting that future opportunities for change were possible, they were more likely to accept and integrate the experiences associated with guilt than with shame. This difference disappeared when the target event was non-repeatable, thereby providing no future opportunities for change. Study 2 further demonstrated the moderating role of future coping confidence in the relationship between the interaction effect of emotion type and event repeatability on self-integration. These findings underscore the different roles of guilt and shame in identity development and intrapersonal learning.

## 1. Introduction

Learning from one’s own experiences, or intrapersonal learning, involves the process of accepting and integrating past selves and events into one’s current self-concept [1,2], resulting in an understanding of the significance and causal meaning of one’s past experiences [3]. Unfortunately, learning from one’s own experiences becomes challenging when the target past event is negative (e.g., failures, mistakes, and wrongdoing), because the learning process in this case requires overcoming fear of the ego threat [4]. When looking back, guilt and shame, which are highly self-conscious emotions, are likely to arise when individuals evaluate themselves as failing to meet their own and others’ standards [5,6,7]. Considering that shame and guilt reflect negative evaluations of oneself but serve self-regulatory functions by providing rich information for future improvement [8], how well would the past events that elicit shame and guilt be integrated into conceptions of the self? This study investigated the extent to which people integrate the past experiences that elicit feelings of guilt or shame into their current self-concept. Although previous research on self-conscious emotions (i.e., guilt and shame) demonstrated that shame and guilt are distinct emotions with regards to their antecedents and consequences (see [9]), there has been little attempt to distinguish the two emotions in the context of self-integration [10]. Given that shame and guilt are highly self-evaluative emotions, it is important to elaborate on the link between self-conscious emotions and the process of the formation of the self-concept. Drawing on the framework of attributional theories in the literature on guilt and shame that argue that guilt has greater implications for future behavioral change than shame [9], it was hypothesized that guilt events would be more likely than shame events to be integrated into one’s self-concept when the event provided a future opportunity for the individual to improve or change the outcome of the event.

### 1.1. Distinguishing between Guilt and Shame

Although guilt and shame are used interchangeably in daily conversations, research has shown that these two emotions are distinct in their cognitive, motivational, and behavioral aspects [9,11]. Regarding the basis of appraisals, guilt is derived from evaluating one’s own erroneous actions, particularly in situations that affect others (e.g., talking behind others’ backs) or failing at duties [12,13], whereas shame arises from perceiving flaws within oneself (e.g., poor performance) [14]. Tracy and Robin [15] illustrated that guilt is often associated with unstable, controllable, and specific aspects of the self that can be corrected (e.g., insufficient effort), whereas shame is linked to stable, uncontrollable, and global aspects of the self that are difficult to alter (e.g., lack of talent). Consequently, guilt has been reported to enhance a sense of control (e.g., [16]). These distinctions suggest that guilt offers a greater likelihood of future behavioral change compared to shame, because modifying particular behaviors is presumably simpler than altering one’s entire character. Conway and Peetz [17] showed that recalling past immoral acts leads individuals to engage in acts of moral recovery (e.g., prosocial behavior) when past immoral behavior is construed at a specific level. Conversely, recalling past wrongdoing leads people to act consistently with those past actions when construed in an abstract way. Another hint about the link between self-conscious emotions and self-integration comes from research investigating the impact of shame and guilt on the way people process and interpret information. For example, Han, Duhachek, and Agrawal [18] demonstrated that guilt leads to low-level construal (see [19]) with a focus on the means of achieving outcomes. In contrast, shame results in high-level construal, emphasizing outcomes over means. Thus, with guilt stemming from a focus on precise and actionable behaviors and shame from a broader evaluation of the self [14], it is evident that experiencing guilt provides a better foundation than experiencing shame for potential future behavioral adjustments [8] and, more importantly, self-integration. When it comes to motivational consequences, guilt leads to action tendencies to repair situations involving others [12,20], whereas shame leads to action tendencies to withdraw from shame-eliciting situations [8,21,22]. Consequently, an individual feeling guilty is inclined to address the situations that trigger guilt, creating an opportunity to amend past wrongs or mend damaged relationships. In contrast, someone experiencing shame may struggle to find opportunities to rectify the situation because their inclination is to avoid or withdraw from it. Indeed, research with young prisoners reported that guilt is negatively associated with recidivism, whereas a feeling of shame predicts higher recidivism [23,24]. Furthermore, such differences between shame and guilt in terms of future behavioral changes are reflected in the inferences people make based on others’ expressions of shame and guilt. For example, Choi [25,26] showed that people expect a greater level of future behavioral change (i.e., the intention to make reparations) from a target person who expresses guilt rather than shame for their misdeeds. In conclusion, the converging evidence shows that, when individuals experience guilt (vs. shame), they have a greater potential to change their wronged past behavior. Given the preceding analysis, it may be worthwhile to ask how well guilt and shame can be integrated into one’s self-concept.

### 1.2. Self-Conscious Emotions and Integration into the Self-Concept

Integration is the process of recognizing and aligning aspects of oneself, such as values, emotions, identities, and beliefs, into a unified whole [27]. Integrating past experiences into one’s self-concept means that an individual feels a sense of continuity and coherence between past experiences and the present self, understands that past experiences are relevant to the present, and accepts the characteristics and events of the past as part of who they are [28,29]. This enables individuals to develop consistent and evolving self-concepts. Research has shown that accepting both a positive and negative past as part of one’s self-concept is important for identity development and personal growth [30,31]. However, people find it challenging to accept and integrate negative past experiences because these threaten their self-image [32]. Of particular interest, given that this study examines the integration of negative past experiences that elicit shame and guilt, this study focuses on the acceptance of a past event as an indicator of self-integration. This study argues that the distinct characteristics of shame and guilt lead to differences in self-integration. Shame results from global self-evaluation, whereas guilt involves the evaluation of specific behavior, suggesting that a person who feels ashamed thinks they have no control over the situation [15]. Such differences in the sense of control and motivational consequences (i.e., avoidance versus approach) between guilt and shame speak to which self-conscious emotion would be more likely to be included in the present self-concept. Interestingly, Tangney et al. [10] showed that the tendency to experience shame is positively associated with both actual–ought and actual–ideal self-discrepancies [33]. However, the proneness to guilt was not associated with these self-guides (i.e., the actual self that represents hopes and wishes and the ought self that represents duties and responsibilities). Consequently, shame, which is the overall evaluation of the flawed self, is more likely to trigger self-protective motivations. Research on temporal self-appraisal [34] points out that people tend to be motivated to distance themselves to minimize the negative implications of the past self on the current self by overestimating the temporal distance between the past self and the current self. Thus, similar to the defense mechanism in temporal self-appraisals, people would be motivated not to include the past ashamed self, because the ugly self in the past carries negative implications for the current self. Instead, an ashamed person may find the target of blame to deflect negative implications [35]. Weinstein, Deci, and Ryan [29] showed that autonomously motivated individuals are more accepting of both positive and negative past aspects, whereas people with controlled motivation distance themselves from negative past identities to protect their self-image. Considering that autonomy is a motivational state of self-initiation, which is based on a good sense of control and behavioral effectance, the findings of Weinstein et al. [29] support the idea that guilt experiences are more likely than shame-eliciting experiences to be integrated into the self-concept. Similarly, Vess, Schlegel, Hicks, and Arndt [36] showed that the participants who thought about the characteristics of the self that they believed to be reflective of who they truly are (i.e., true self-concept; [37]) increased their (shame-free) guilt and decreased their (guilt-free) shame for negative evaluative experiences (e.g., poor performance). It is important to note that the buffering effect of the concept of the true self in response to self-threatening information held true only for guilt, suggesting that guilt is more likely than shame to be included in one’s self-concept when an individual feels safe with regard to maintaining one’s positive self-image.

### 1.3. Future Opportunity

This study introduces a future opportunity as a potential moderator that may influence the manner in which guilt and shame are integrated into one’s self-concept. With its action-oriented nature, guilt is more readily accepted and integrated when individuals perceive opportunities for future rectification. This process is facilitated by the forward-looking nature of guilt, which is characterized by future behavioral changes. The presence of future opportunities to change the outcome of an event in the past may attenuate the defensive motivation, because the outcome could be less threatening (cf. [38]). Indeed, research on regret and counterfactual thinking has shown that future opportunities mitigate regret intensity [39] and interrupt the activation of the psychological immune system, which reflects self-protective motivation (e.g., [40]). Connecting shame and guilt to the status of future opportunities, previous research showed that guilt-prone people are more likely to consider distant future consequences, whereas shame-prone people are less likely to consider the consequences of current actions that are distant in the future [41], suggesting that guilt is more sensitive than shame to future opportunities. Recently, Choi [25] suggested that placing guilt and shame in a future-relevant context is an effective way to distinguish the subtle differences between guilt and shame in the implications for future changes. Shame would not benefit from having future opportunities, because it would not matter for a shameful individual to have such an opportunity. A person who is ashamed believes that the entire self is ruined and that the flawed self cannot be repaired. However, guilty events can be included in self-construal to a greater extent. This is because the event is less threatening as long as there is a second chance to correct mistakes and wrongdoing. Thus, under the conditions of high future opportunities (i.e., repeatable events), the past event that elicited guilt seems more reflective of oneself, indicating self-integration. Considering that guilt results from the appraisal of one’s wronged behavior that can be easily changed in the future, whereas shame results from the evaluation of defects in the overall self that are hardly changed [7], it was hypothesized that people would be more likely to accept their past guilt events as part of their self when the past event provides a future opportunity to change the outcome of the event (e.g., a repeatable event). This is not the case with past shame events. Guilt events no longer imply negative images of the self when past wrongdoings can be corrected. However, when there is little or no chance to correct past outcomes (i.e., an event is non-repeatable), guilt and shame are not distinguished in the self-integration process.

## 2. Study 1

### 2.1. Method

#### 2.1.1. Participants and Design

A total of 201 participants from an online survey website (Amazon Mechanical Turk) participated in Study 1 (*M*_age_ = 35.74, *SD* = 13.09; 53.2% female; 75.6% Caucasian, 8% African American, 7% Asian/Asian American, 6% Hispanic/Latino, and 3% other). The participants were randomly assigned to the conditions of a 2 (emotion type: guilt vs. shame) × 2 (event repeatability: repeatable vs. non-repeatable) between-participants design (*n* = 49~52 per cell). A sensitivity power analysis employing GPower (*N* = 201, α = 0.05, two-tailed, and power = 80%) revealed that the sample was sufficiently powered to detect a minimum effect size of *f* = 0.24.

#### 2.1.2. Procedure and Materials

The participants were asked to recall and describe a target event in response to a prompt according to their assigned conditions. Following previous research (e.g., [42,43]), this study manipulated the event repeatability to operationalize perceived future opportunities. A repeatable event is perceived as having a high opportunity to change outcomes in the future, whereas a non-repeatable event is perceived as having a low opportunity to do so. The participants in the repeatable shame condition read the following instructions:

*Try to recall an outcome or event from your past that made you feel ashamed. The event outcome that you recall should be one that you could potentially improve upon in the future. In other words, the event outcome that you choose to recall should be one that could possibly happen to you again in the future. For example, you may have experienced shame in the past if you performed poorly on a presentation in front of your classmates or colleagues and you expect that you will be giving similar presentations in the future, or if you hurt the feelings of a friend whom you expect to see again.*

The participants in the non-repeatable shame condition read the following instruction:

*Try to recall an outcome or event from your past that made you feel ashamed. The event outcome that you recall should be one that you cannot improve upon in the future. In other words, the event outcome that you choose to recall should be one that will probably not happen to you again in the future. For example, you may have experienced shame in the past if you performed poorly on a presentation in front of your classmates or colleagues and you do not expect to be giving similar presentations in the future, or if you hurt the feelings of a friend whom you do not expect to see again.*

The participants in the repeatable guilt event condition read the following instruction:

*Try to recall an outcome or event from your past that made you feel guilty. The event outcome that you recall should be one that you could potentially improve upon in the future. In other words, the event outcome that you choose to recall should be one that could possibly happen to you again in the future. For example, you may have experienced guilt in the past if you neglected your duties as a member of a team that was working on an ongoing project, or if you lied to a friend whom you expect to see again.*

The participants in the non-repeatable guilt event condition read the following instruction:

*Try to recall an outcome or event from your past that made you feel guilty. The event outcome that you recall should be one that you cannot improve upon in the future. In other words, the event outcome that you choose to recall should be one that will probably not happen to you again in the future. For example, you may have experienced guilt in the past if you neglected your duties as a member of a team that was working on a one-time project, or if you lied to a friend whom you do not expect to see again.*

After completing the event description task, the participants rated the intensity of guilt and shame (“*Indicate the extent to which you were feeling each of the following emotions while you were recalling and writing about the event*”) using a 10-point scale (1 = *not at all*, 7 = *very strongly*). Then, the participants rated the degree of character integration (*“To what extent do you think the event reflects the true nature of yourself?”*) on a 7-point scale (1 = *not at all*, 7 = *very much*). Being connected to the (true) attributes of one’s past experiences means that the past is well integrated into one’s self-concept [1]. Thus, this study employed the sense of the true self that the participants felt towards the past self as an index of self-integration (i.e., character integration). Finally, to guarantee that the participants correctly recalled the target event with regards to the degree of repeatability (i.e., repeatable vs. non-repeatable), the participants rated how much they believed the event would have a future opportunity on five items (e.g., *“I will certainly have opportunities for positive action in incidents similar to the one I reported”*) [42] using a 7-point scale (1 = *not at all*, 7 = *very much*).

### 2.2. Results

#### 2.2.1. Manipulation Check

To confirm that the participants in the shame condition recalled a shame-associated event, a 2 (*emotion type*: guilt vs. shame) × 2 (*event repeatability*: repeatable vs. non-repeatable) analysis of covariance (ANCOVA), with the guilt intensity rating as a covariate performed on the guilt-free shame intensity rating, revealed a significant main effect of the emotion type (*F*(1, 185) = 20.83, *p* < 0.001, and η_p_^2^ = 0.101), indicating that the participants in the shame condition reported a greater level of shame (*M* = 6.59, *SD* = 2.75), compared to those in the guilt condition (*M* = 5.64, *SD* = 2.96). The same ANCOVA, with the shame intensity rating as a covariate performed on the shame-free guilt intensity rating, revealed a significant main effect of the emotion type (*F*(1, 185) = 15.26, *p* < 0.001, and η_p_^2^ = 0.076), indicating that the participants in the guilt condition (*M* = 6.24, *SD* = 2.84), compared to those in the shame condition (*M* = 6.09, *SD* = 2.93), rated higher on guilt intensity. There were no significant interaction effects in either analysis (*F*s < 0.05).

To check whether the participants followed the instruction regarding event repeatability, a 2 (*emotion type*: guilt vs. shame) × 2 (*event repeatability*: repeatable vs. non-repeatable) analysis of variance (ANOVA) was conducted on the mean ratings of future opportunity (Cronbach’s *α* = 0.775). The analysis yielded no significant interaction effect (*F*(1, 186) = 0.03, *p* = 0.865, and η_p_^2^ = 0.000). However, there was a significant main effect of event repeatability (*F*(1, 186) = 38.16, *p* < 0.001, and η_p_^2^ = 0.17), indicating that participants in the repeatable condition (*M* = 4.05, *SD* = 1.39) recalled the event that provided a greater level of future opportunity than did those who were in the non-repeatable condition (*M* = 2.83, *SD* = 1.34).

Thus, the manipulations of the emotion type and event repeatability were successful.

#### 2.2.2. Character Integration

**Figure 1:**
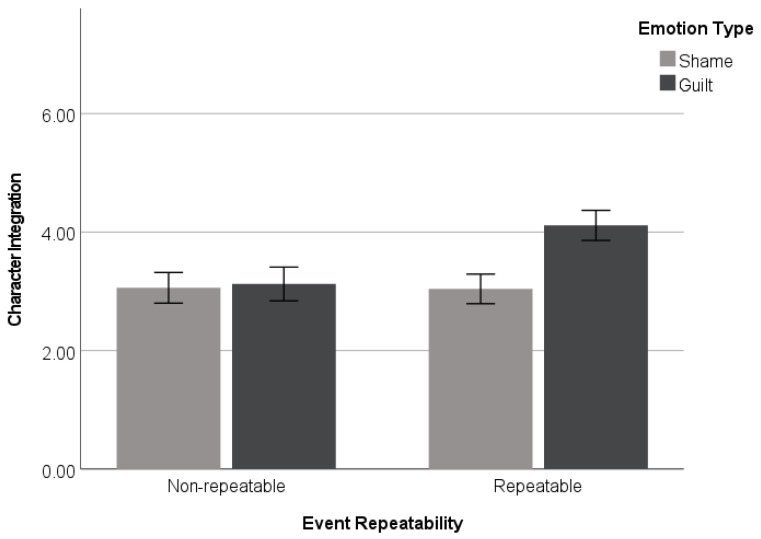
Character integration as a function of emotion (guilt vs. shame) × event repeatability (repeatable vs. non-repeatable) (Study 1). Error bars represent +/− 1 standard errors.

To test the hypothesis that guilt-associated events are more likely to be integrated into the self when the target event is repeatable, a 2 (*emotion type*: guilt vs. shame) × 2 (*event repeatability*: repeatable vs. non-repeatable) ANOVA was performed on character integration. The analysis revealed a marginally significant emotion type × event repeatability interaction effect (*F*(1, 186) = 3.66, *p* = 0.057, and η_p_^2^ = 0.019). Of particular interest, simple effects analyses were then conducted to explore the primary hypothesis. Consistent with the predictions, the participants in the guilt event condition (*M* = 4.11, *SD* = 1.69) reported higher than those in the shame event condition (*M* = 3.04, *SD* = 1.73) on the ratings of character integration when the event is believed to be repeatable (*F*(1, 186) = 7.72, *p* = 0.006). On the other hand, when the event was believed to be non-repeatable, there was no such difference in character integration between the guilt (*M* = 4.11, *SD* = 1.69) and shame event conditions (*M* = 3.04, *SD* = 1.73), with *F*(1, 186) < 1 (see Figure 1).

### 2.3. Discussion

Study 1 tested the interplay between self-conscious emotions and future opportunities (i.e., event repeatability) on self-integration. It was hypothesized that, because guilt has greater implications for future behavioral change compared to shame, the participants who recalled guilt (vs. shame) experiences would be more likely to integrate the target event into their self-concept when they believed that the event provided a future opportunity for change (i.e., repeatable). It was also hypothesized that the difference between guilt and shame would be muted when the event had no future opportunity for change (i.e., non-repeatable). Supporting this hypothesis, the results showed that the participants in the guilt condition rated higher than those in the shame condition on character integration only when it was believed that the event could be repeated. These results indicate that both the presence of future opportunities and the future-oriented self-conscious emotion (i.e., guilt) are conducive to accepting negative past experiences as part of the self, which could lead to the development of one’s self-concept and personal growth. However, Study 1 did not provide a detailed mechanism by which past guilt and shame experiences are connected or disconnected from the current self-understanding. Furthermore, given that Study 1 used a single item to measure character integration, the interpretation of its findings is limited. Study 2 attempted to address these issues.

## 3. Study 2

In addition to character integration (i.e., acknowledging the connection between the target event and the concept of the true self), Study 2 measured event integration (i.e., the extent to which people accepted a past life event) from Weinstein et al. [29]. Furthermore, Study 2 explored the possible moderating effect of future coping confidence on the interaction effect of emotion type and event repeatability, as reported in Study 1. It was hypothesized that the greater the participants’ feelings of guilt, the higher their self-integration, particularly when the event was viewed as repeatable and their future coping confidence was high. Conversely, such differences would disappear when the event was viewed as repeatable but future coping confidence was low. The rationale was that future opportunities do not guarantee that corrective actions in the future will be successful. Thus, an individual must be assured that there is both an opportunity to correct past mistakes and that they know how to cope with similar situations in the future. Research has shown that health intervention program messages are effective in inducing health-promoting behaviors when threat-based information (e.g., breast cancer) is presented together with ways to avoid such threats [44]. Similarly, guilt- and shame-eliciting experiences threaten self-image; therefore, the experiences need to be accompanied by ways to avoid or cope with such aversive situations when future occurrences are highly likely. Consequently, Study 2 included future coping confidence to elaborate the links between self-conscious emotions, event repeatability, and self-integration.

### 3.1. Method

#### 3.1.1. Participants and Design

A total of 221 respondents from an online survey website (Amazon Mechanical Turk) participated in Study 2 (*M*_age_ = 35.07, *SD* = 11.16; 66.1% female; 76% Caucasian, 8.1% African American, 7.2% Asian/Asian American, 3.6% Hispanic/Latino, and 2.3% other). The participants were randomly assigned to the conditions of a 2 (emotion type: guilt vs. shame) × 2 (event repeatability: repeatable vs. non-repeatable) between-participants design (*n* = 52~58 per cell). A sensitivity power analysis employing GPower (*N* = 221, α = 0.05, two-tailed, and power = 80%) revealed that the sample was sufficiently powered to detect a minimum effect size of *f* = 0.22.

#### 3.1.2. Procedure and Materials

The procedure was identical to that used in Study 1, except for some changes in the measures. First, in addition to the character integration item in Study 1, Study 2 included event integration measures (i.e., “*I accept the experience I had*” and “*I embrace that this event is a part of my past*”) [29] to capture the various aspects of the integration into one’s self-concept. Furthermore, the future opportunity perception measures were simplified and narrowed down to one question about the perceived likelihood of a target event happening again in the future. Finally, at the end of the experimental session, the participants in Study 2 were asked to indicate their confidence in coping with future events similar to the target event they recalled.

### 3.2. Results

#### 3.2.1. Manipulation Check

As in Study 1, 2 (*emotion type*: guilt vs. shame) × 2 (*event repeatability*: repeatable vs. non-repeatable) ANCOVAs were conducted on the guilt and shame intensity ratings. First, an ANCOVA with the guilt intensity rating as a covariate performed on the shame intensity rating (i.e., guilt-free shame) revealed a significant main effect of the emotion type (*F*(1, 196) = 36.38, *p* < 0.001, and η_p_^2^ = 0.157), indicating that the participants in the shame condition (*M* = 8.77, *SD* = 1.76), compared to those in the guilt condition (*M* = 7.50, *SD* = 2.54), reported a greater level of shame. The same ANCOVA with the shame intensity rating as a covariate performed on the guilt intensity rating (i.e., shame-free guilt) revealed a significant main effect of the emotion type (*F*(1, 196) = 19.99, *p* < 0.001, and η_p_^2^ = 0.093), indicating that the participants in the guilt condition (*M* = 8.03, *SD* = 2.16), compared to those in the shame condition (*M* = 7.58, *SD* = 2.79), reported a greater level of guilt. There were no significant interaction effects in either analysis (*F*s < 0.86).

To confirm that the participants followed the instructions regarding event repeatability, a 2 (*emotion type*: guilt vs. shame) × 2 (*event repeatability*: repeatable vs. non-repeatable) ANOVA was conducted on the likelihood rating. The analysis yielded an unexpected significant interaction effect (*F*(1, 197) = 4.51, *p* = 0.035, and η_p_^2^ = 0.022). Further analysis showed that the significant effect was driven by the mean difference between guilt (*M* = 7.37, *SD* = 2.35) and shame (*M* = 6.08, *SD* = 2.26) in the repeatable event condition (*F*(1, 197) = 10.56, *p* = 0.001), suggesting that the participants viewed guilt-associated events as having a greater opportunity to change outcomes in the future. There was no such difference between the two conditions in the non-repeatable event condition (*M_Guilt_* = 2.17, *SD* = 1.82; *M_Shame_* = 2.09, *SD* = 1.44), with *F*(1, 197) < 0.2. This finding is interesting because it shows the very nature of guilt experiences regarding future change. It appears that likelihood estimation is a more sensitive measure of the subjective experience of a target event regarding the status of future opportunities. The main effect of event repeatability was significant (*F*(1, 197) = 262.11, *p* < 0.001, and η_p_^2^ = 0.571), indicating that participants in the repeatable condition (*M* = 6.73, *SD* = 2.39) recalled the event that provided a greater level of future opportunity than did those who were in the non-repeatable condition (*M* = 2.13, *SD* = 1.65).

#### 3.2.2. Character Integration

**Figure 2:**
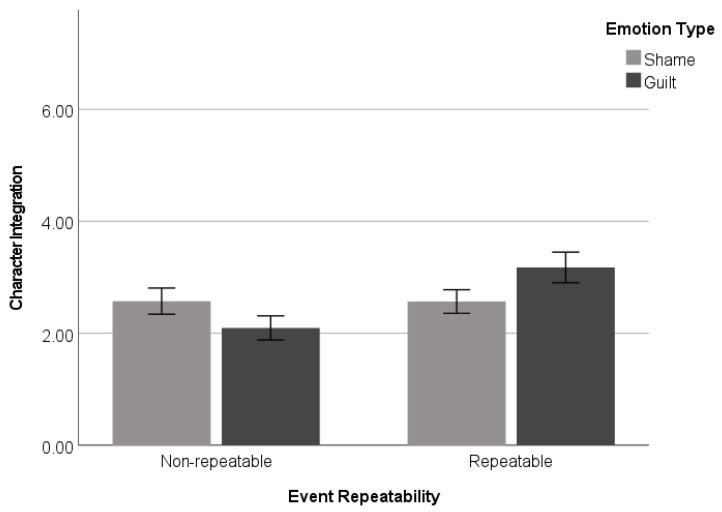
Character integration as a function of emotion (guilt vs. shame) × event repeatability (repeatable vs. non-repeatable) (Study 2). Error bars represent standard errors.

As in Study 1, a 2 (*emotion type*: guilt vs. shame) × 2 (*event repeatability*: repeatable vs. non-repeatable) ANOVA was performed on character integration. The analysis revealed a significant emotion type × event repeatability interaction effect (*F*(1, 197) = 5.36, *p* = 0.022, and η_p_^2^ = 0.026). Simple effects analyses were conducted to test the primary hypothesis. Replicating the findings of Study 1 and consistent with the predictions, when participants believed that the target event was repeatable, they rated character integration higher for guilt-associated events (*M* = 3.18, *SD* = 1.96) than for shame-associated events (*M* = 2.57, *SD* = 1.50) (*F*(1, 197) = 3.41, *p* = 0.066), although the effect was only marginally significant. In contrast, the participants in the non-repeatable condition did not show such a difference between guilt-associated events (*M* = 2.10, *SD* = 1.55) and shame-associated events (*M* = 2.57, *SD* = 1.60) (*F*(1, 197) = 2.29, *p* = 0.132) (see Figure 2).

#### 3.2.3. Event Integration

**Figure 3:**
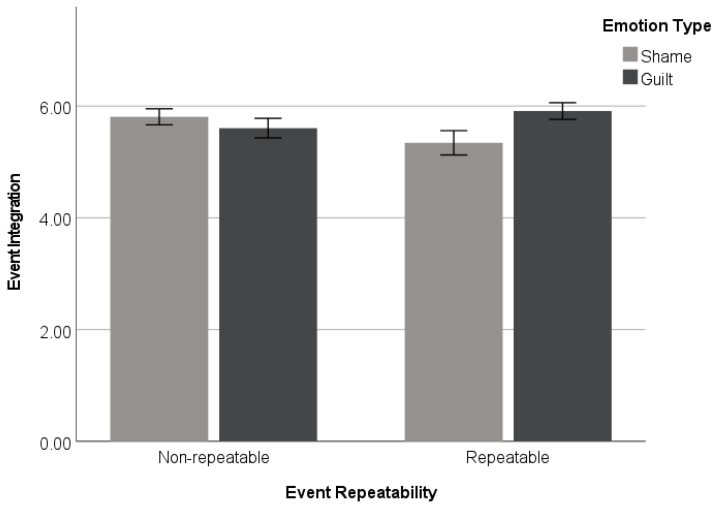
Event integration as a function of emotion (guilt vs. shame) × event repeatability (repeatable vs. non-repeatable) (Study 2). Error bars represent +/− 1 standard errors.

The event integration index was computed by averaging the two items: *r*(199) = 0.56 and *p* < 0.001. To test the hypothesis that the participants in the guilt-associated (vs. shame-associated) event condition were more likely to accept past events as part of the self when the event was viewed as repeatable (but not when the event was believed to be non-repeatable), a 2 (*emotion type*: guilt vs. shame) × 2 (*event repeatability*: repeatable vs. non-repeatable) ANOVA was performed on the event integration ratings. The analysis revealed a significant emotion type × event repeatability interaction effect (*F*(1, 197) = 4.83, *p* = 0.029, and η_p_^2^ = 0.024). In line with the mean difference patterns of character integration and consistent with the predictions, a simple effects analysis showed that the participants in the repeatable event condition reported higher ratings of event integration for guilt-associated events (*M* = 5.91, *SD* = 1.06) than shame-associated events (*M* = 5.34, *SD* = 1.56) (*F*(1, 197) = 5.33, *p* = 0.022). In contrast, the participants who recalled non-repeatable events showed no such difference between guilt-associated events (*M* = 5.61, *SD* = 1.27) and shame-associated events (*M* = 5.81, *SD* = 0.99), with *F*(1, 197) < 1 (see Figure 3). These results show that guilty experiences are more likely to be integrated into the current self-concept as long as future opportunities to correct past mistakes are open compared to the experiences that elicited shame.

#### 3.2.4. Future Coping Confidence

Study 2 further explored the possible moderating effect of future coping confidence on the interaction effect of emotion type and event repeatability reported in Study 1, such that the greater the participants’ feelings of guilt, the higher their self-integration, particularly when the event was viewed as repeatable and future coping confidence was high. Conversely, such differences would disappear when the event was viewed as repeatable but future coping confidence was low. To test this idea, model 2 was used in the PROCESS 4.3 macro for SPSS [45]. The results revealed that the unconditional interaction of emotion type and event repeatability was not significant (β = 0.533, *t* = 1.61, and *p* = 1.09). The unconditional interaction of emotion type and future coping confidence was significant (β = 0.237, *t* = 3.62, and *p* < 0.001). Simple slope tests were performed to examine whether there were any conditional interactions between the variables. The results showed that when the event was repeatable and future coping confidence was high, emotion type positively predicted event integration (β = 0.86, *t* = 3.18, *p* < 0.002, and 95% CI [0.32, 1.39]), indicating that the greater the participants’ feelings of guilt, the higher their self-integration. However, when the event was repeatable and future coping confidence was low, emotion type negatively predicted event integration (β = −0.88, *t* = −3.20, *p* < 0.002, and 95% CI [−1.43, −0.39]), indicating that the greater the participants’ feelings of guilt, the lower their self-integration.

### 3.3. Discussion

Replicating the findings of Study 1, Study 2 demonstrates that guilt events are more likely to be integrated into the self-concept when people believe that the event will happen again in the future. Importantly, the main findings hold true only when participants felt confident in coping with a likely future event, indicating that one’s defense mechanism plays a significant role in the link between highly self-evaluative emotions (i.e., guilt and shame) and the self-concept.

## 4. General Discussion

This study aims to delineate the relationship between self-conscious emotions and self-integration. By manipulating the types of self-conscious emotions (i.e., guilt versus shame) and future opportunities (repeatable event versus non-repeatable event), this study demonstrated that guilt than shame leads to a greater level of self-integration when there is a future opportunity to change the outcome (Study 1), especially for those who feel confident in coping with the future situation (Study 2). The consistent findings of the current study indicate that guilt has a greater potential than shame for personal growth and psychological well-being. This study contributes to the extant literature on emotions by exploring a relatively understudied subtopic, namely, the link between self-conscious emotions and the process of self-integration. This study demonstrates the constructive potential of guilt in fostering change and adaptation, underscoring the importance of distinguishing between the emotions of shame and guilt in the formation of the self-concept. Furthermore, the findings of this study align with the main findings on the human learning process in a broader context. Although people think they learn from negative life events that elicit negative emotions [46], they learn less from failure than from success [4]. However, self-conscious emotions are unique as they are highly self-evaluative, serving both self-regulatory functions [6] and social functions [47,48]. In this sense, understanding the interplay between self-conscious emotions, such as guilt and shame, and their integration into the self-concept, particularly in the context of future opportunities, presents a significant question regarding intrapersonal and interpersonal learning. By situating shame and guilt in a future-specific context [25,26], this study distinguishes the implications of guilt and shame for self-integration and identity development. The role of future opportunities in the integration process highlights the dynamic nature of the self-concept, which is not only reflective of past experiences but also shaped by the anticipation of future selves (e.g., [49]). This forward-looking aspect of the integration into one’s self-concept emphasizes the importance of temporal perspectives in understanding how self-conscious emotions influence identity formation (cf. [50]). The implications of integrating guilt versus shame into the self-concept extend beyond personal growth to include social relationships and moral behavior. Guilt’s reparative orientation not only facilitates personal development but also strengthens social bonds through the acknowledgment of the harm impacting others and the initiation of amends. By contrast, the isolating nature of shame can lead to social withdrawal and a breakdown in relationships, further complicating the integration process. One caveat is that the current findings may reflect people’s expectations or a normative understanding of self-conscious emotions. People often believe that they have learned from experiences that elicit negative emotions, which may not be the case [46]. Thus, future research should measure actual learning or performance improvement (e.g., social skills) as a result of the integration into one’s self-concept. Furthermore, owing to the challenges raised by the complex and dynamic nature of the self [51], this study investigated, as a first step, only one aspect of the development of the self-concept with regard to the experiences of guilt and shame. Extending the current investigation to narrative identity could further elucidate the link between self-conscious emotions and the self-concept [52]. Additionally, there may be situations where shame is more conducive than guilt to integrating the past into the current self-concept over the long term (cf. [53]).

## 5. Conclusions

Self-conscious emotions such as guilt and shame play pivotal roles in the development of and the integration into one’s self-concept. This study suggests that having future opportunities can help people better integrate past guilt experiences into their self-concept compared to past shame experiences, because guilt is believed to have a greater potential for future change compared to shame. This study provides valuable insights into how individuals experience and process different self-conscious emotions and their impact on future behavior and self-perception.

## Data Availability

The data presented in this study are available upon request from the corresponding author.
